# Field‐Collected Belgian *Culex pipiens* and *Culex torrentium* Mosquitoes are Competent for West Nile Virus Under Different Temperature Conditions

**DOI:** 10.1155/tbed/4759838

**Published:** 2026-09-21

**Authors:** Evi Brams, Charlotte Sohier, Nick De Regge

**Affiliations:** ^1^ Sciensano, Exotic- and Vector-Borne Diseases, Groeselenbergstraat 99, Brussels 1180, Belgium, sciensano.be

**Keywords:** Belgium, *Culex pipiens*, *Culex torrentium*, field-collected mosquitoes, transmission, vector competence, West Nile virus

## Abstract

Belgium detected West Nile virus (WNV) for the first time on its territory in August 2025 during a monitoring of wild birds. To support risk assessments, we determined the vector competence of field‐collected Belgian *Culex pipiens* and *Culex torrentium* mosquitoes, which are considered important WNV vectors. Females fed on a WNV‐spiked (lineage 1 Israel 98 strain) blood meal were kept for 14 days at (i) a constant 25°C, (ii) a 25/15°C day/night gradient reflecting current Belgian summers, or (iii) a 25/20°C gradient simulating a climate change scenario. After the 14‐day incubation period, abdomens, legs/wings/head, and saliva were collected and tested in RT‐qPCR to determine the infection, dissemination, and transmission efficiencies (TEs). Observed infection efficiencies (IEs) (25.5%, 32.7%, and 34.3%) did not significantly differ between temperature conditions, and mostly mosquitoes with the lowest viral loads were dissemination‐positive. The dissemination efficiency (DE) at 25/15°C (28.6%) was higher than in both other conditions and statistically significant compared to 25/20°C (10.4%), indicating that WNV more easily passes the midgut barrier under current Belgian summer temperatures. The entry of WNV into the salivary glands did not seem to be altered by temperature, as no significant differences were observed between the obtained TEs (3.9%, 8.2%, and 1.5% at 25°C, 25/15°C, and 25/20°C, respectively). By performing virus isolations on saliva, we proved that *Culex pipiens* is a competent vector for WNV at 25 and 25/15°C, while this was not confirmed at 25/20°C. Notably, a small percentage of the field‐collected mosquitoes in this study were identified as *Culex torrentium* (7%), which also showed high competence for WNV. To conclude, our results suggest that field‐collected Belgian *Culex pipiens* mosquitoes are competent vectors for WNV at 25°C and under current Belgian temperatures, emphasizing their potential to contribute to local WNV circulation and the importance of ongoing surveillance in birds, horses, and mosquitoes.

## 1. Introduction

West Nile virus (WNV) is a mosquito‐borne virus that belongs to the genus *Orthoflavivirus* [1, 2] and family Flaviviridae. This zoonotic virus has a transmission cycle between mosquitoes and birds [3], including birds of prey and crows [4, 5]. Birds act as a natural reservoir for WNV and can develop high viremia levels, allowing WNV to be transmitted to mosquitoes that feed on infected birds. Humans and horses can also become infected with the virus when bitten by an infected mosquito. However, they are considered dead‐end hosts as virus levels are insufficiently high to allow WNV transmission [6]. Not all bird species develop symptoms of a WNV infection [5], although susceptible species can display neurological signs, including the inability to fly and leg paralysis [7]. In humans, 20%–30% of WNV‐infected individuals will develop symptoms including fever and headache, which is referred to as West Nile fever. Furthermore, 1% of clinical patients will present with severe neurological symptoms, including encephalitis and meningitis, with a mortality rate of around 10% [5, 8].

Since its first detection in 1937 in Uganda, WNV has been established across multiple continents, including Europe [7, 9]. Belgium’s neighboring countries have reported locally acquired infections, with cases first documented in France in 1962 [10] and recurring in 2000 [11, 12]. In 2018, Germany reported its first cases [10], and yearly cases in humans and animals have been reported since then [10, 13–18]. Recently, WNV was introduced in the Netherlands in 2020 and has since been detected in birds in 2022 and in horses in 2025 [10, 19]. Both lineages 1a (France) and 2 (Germany and the Netherlands) strains have been reported in Belgium’s neighboring countries [10, 20, 21], yet it is lineage 2 strains that are responsible for the most recent cases in Europe [22, 23], despite the ongoing cocirculation of both lineages [10]. In Belgium, no locally acquired human or equine WNV cases have been reported to date. However, in August of 2025, WNV (lineage 2) was detected for the first time in wild birds, confirming that the virus had recently been introduced into the country [19, 24, 25]. Given the recent detection of WNV in wild birds, understanding the characteristics of the local mosquito vectors becomes essential for evaluating the risk of onward transmission in Belgium.

*Culex* (*Cx*.) *pipiens* is the most abundant mosquito species in Belgium and Europe [26]. Two different biotypes can be distinguished for *Cx*. *pipiens*, more specifically biotype *pipiens* (Linnaeus 1758) and biotype *molestus* (Forskål 1775). These biotypes are morphologically identical, though they display different behavior [27, 28]. Eggs and larvae of *Cx*. *pipiens* biotype *pipiens* are mostly found in aboveground habitats like standing water in artificial containers, and adults may prefer birds for blood feeding. On the contrary, *Cx*. *pipiens* biotype *molestus* is mainly associated with underground environments, such as basements and sewers. This biotype is also more inclined to feed on mammals, including humans, and shows autogeny, that is, the ability to lay eggs without a blood meal [27, 29–31]. Interbreeding of both biotypes can occur, leading to hybrids with intermediate host‐feeding behavior [29, 32]. Another common mosquito species that often occurs together with *Cx. pipiens* is *Cx. torrentium* (Martini 1925). Female mosquitoes of this species are morphologically indistinguishable from *Cx. pipiens*. Furthermore, *Cx. torrentium* mosquitoes share their breeding and blood feeding preferences with *Cx. pipiens* [33–36].

*Cx. pipiens* is considered the most important WNV vector as this species has been proven vector‐competent in a laboratory environment and found positive for WNV in the field. Vector competence is a crucial factor in determining the role of *Cx*. *pipiens* in the transmission of WNV as it reflects the intrinsic ability of a mosquito to become infected with a pathogen and to transmit this pathogen to a new host [37]. So far, vector competence of *Cx. pipiens* (including biotypes and hybrids) for WNV has been experimentally demonstrated in field‐collected mosquitoes or laboratory colonies from Belgium, France, Italy, the Netherlands, Germany, and Spain [27, 28, 38–42]. Additionally, *Cx. pipiens* is a high‐capacity vector due to its feeding preferences and high abundance in Belgium and Europe.

Vector competence and vectorial capacity are highly influenced by temperature, as temperature affects many important factors of a mosquito’s life cycle [9, 43] and arbovirus replication dynamics [9]. Several studies have shown that the vector competence of *Cx. pipiens* for WNV increases at higher temperatures [40, 44]. Higher temperatures are also associated with a higher survival rate, development rate, and feeding activity of mosquitoes [45, 46], as well as a shortened extrinsic incubation period [47, 48]. However, too high temperatures may be detrimental to mosquito survival [49] and fecundity [50] and overall WNV establishment in Europe [51].

In this study, we assessed the vector competence of field‐collected Belgian *Cx. pipiens* mosquitoes and studied the impact of temperature thereon. Three temperature conditions were tested: first, a constant temperature of 25°C was chosen based on empirical studies and trait‐based approaches that have shown the thermal optimum of WNV in *Cx. pipiens* is 24.5°C [52, 53]. In addition, 25°C is a commonly used temperature in other vector competence studies, allowing for a comparison of the transmission capability of our mosquito population with that of other mosquito populations. Second, a temperature gradient of 25°C during daytime and 15°C during nighttime (25/15°C) was applied to assess the effect of current Belgian summer temperatures on vector competence. The day and night temperatures of this condition are very similar to the mean maximum and minimum temperatures registered in Belgium during the summer of 2025, which were 24.3°C and 14.2°C, respectively [54]. Third, a temperature gradient of 25°C during daytime and 20°C during nighttime (25/20°C) was tested to study the effect of climate change on vector competence. The Belgian Meteorological Institute namely predicts that the average summer day temperature of 22.5°C will increase to 25.5–27.5°C, and the average summer night temperature will rise from 13.4 to 18.4°C under the highest greenhouse gas emission scenario [55].

## 2. Materials and Methods

### 2.1. Mosquito Collection

Larvae and pupae of *Cx. pipiens* were collected from different water bodies found in Belgium during the vector season (April–November) of 2023 and 2024. The water bodies included rain barrels, ponds, and artificial containers in forests and gardens located in urban and rural areas [56] in the provinces of Antwerp and Walloon Brabant. Larvae and pupae were collected using small fishnets and transported in plastic containers with water from the collection site. In the lab, collected larvae were transferred to plastic trays (39.5 cm × 25.5 cm × 8.8 cm) (AVA, Belgium) containing rainwater and kept at 25°C, 80% relative humidity (RH), and a 15:9 h light–dark cycle (constant climate chamber HPP750eco, Memmert, Germany). To allow the emergence of adult mosquitoes, pupae were transferred to a smaller plastic container (11.5 cm × 11.5 cm × 7 cm) with rainwater, which was placed inside a 24 cm × 24 cm × 24 cm mesh cage (BugDorm, MegaView Science Co., Taiwan) and kept at the same parameters as above. Three times per week, a pinch of larval food (200 mg/L) was added to each larval tray and consisted of a 1:1:1 ratio of Good Pearls koi fish food (Tom & Co., Belgium), liver powder (Q&Q Baitproducts, the Netherlands), and CuniAdult complete rabbit food (Versele‐Laga, Belgium). Pupae were transferred to the emergence cage three times per week. Emerged adults were placed in a separate 24 cm × 24 cm × 24 cm mesh cage, and a 10% sucrose solution was provided ad libitum.

### 2.2. Virus Production and Titration

All experiments were performed using a ninth passage of the WNV lineage 1 Israel 98 strain on Vero cells. Cells were kept at 37°C in Dulbecco’s Modified Eagle’s Medium (DMEM) (Gibco, United States) containing 0.2% gentamycin (Gibco, United States) and amphotericin B (Gibco, United States) and 5% fetal bovine serum (FBS) (Life Technologies Europe, the Netherlands). At 72 h postincubation and after three freeze–thaw cycles, the supernatant was collected and stored at −80°C. The titer of the stock was determined by virus titration on Vero cells and was calculated to be 10^6.2^ TCID_50_/mL.

### 2.3. Oral Infection of Mosquitoes

Three to 14‐day‐old F0‐generation *Cx. pipiens* mosquitoes were deprived of any food and water source 48 h before exposure to the blood meal and transferred to the infection cage 24 h before exposure. On the day of infection, the mosquitoes were transported to the BSL‐3 facilities and placed in a cage‐in‐cage‐in‐cage system, consisting of the 11.5 cm × 11.5 cm × 7 cm infection cage inside a 32.5 cm × 32.5 cm × 32.5 cm cage inside a 93 cm × 47.5 cm × 47.5 cm cage (BugDorm, MegaView Science Co., Taiwan). Mosquitoes were offered a WNV‐spiked blood meal, with a titer of 10^5.7^ TCID_50_/mL, for 1 h using the Hemotek feeding system (Hemotek, United Kingdom) covered with a pig intestine membrane (butcher shop Somers, Boechout, Belgium). The blood was heated to 37°C, and the temperature was regularly checked. Chicken blood (poultry slaughterhouse Van‐O‐Bel, Waregem, Belgium) was used for preparing the infectious blood meal and defibrinated at the time of collection by adding the blood to an equal amount of Alsever’s solution (Sigma–Aldrich, United States). The blood was supplemented with adenosine triphosphate (VWR, United States) at a 100:1 ratio (29.7 µg per mL of blood) and WNV stock at a 2:1 ratio. Back titrations showed that the virus titer decreased by approximately 1 log during the 1‐h blood feeding period. After blood feeding, the mosquitoes were cold‐anesthetized at −20°C for 2 min and transferred to a glass petri dish on ice. Blood‐fed females were selected, transferred to a 29 cm × 29 cm × 29 cm plastic cage (BugDorm, MegaView Science Co., Taiwan), and kept at either a constant 25°C or at a gradient (day/night) temperature of 25/15 or 25/20°C with a RH of 80% and a 15:9 h light–dark cycle (constant climate chamber HPP750eco, Memmert, Germany) for 14 days, with unlimited access to a 10% sucrose solution for 12 days. We aimed to obtain 50 blood‐fed mosquitoes surviving the 14‐day incubation period per temperature condition.

### 2.4. Dissection and Salivation

Blood‐fed mosquitoes were deprived of food and water at 12 days post‐blood feeding. After the 14‐day incubation period, the surviving mosquitoes were captured in 15 mL Falcon tubes (Corning, United States) and placed on ice for immobilization. The wings and legs were removed with tweezers and added to a 2 mL EXPell Secure microcentrifuge tube (CAPP, Germany) containing 500 µL of DMEM and 2% antibiotic–antimycotic (AA) solution (Gibco, United States). After this, mosquitoes were fixed to a microscopic slide (Glaswarenfabrik Karl Hecht, Germany) using double‐sided tape (Tesa, Germany), and the proboscis was inserted into a 20 µL pipette tip containing 5 µL of FBS with 10% sucrose, which was held in place by plasticine. Salivation was evaluated by indicating the level of FBS in the pipette tip before salivation and assessing the level after salivation. A decrease in the volume of FBS indicates that the mosquito has fed on FBS and salivated into the pipette tip. Additionally, abdominal swelling was assessed visually after salivation. Mosquitoes were allowed to salivate in the dark for 15 min, after which the content of the pipette tip was added to a 1.5 mL EXPell Secure microcentrifuge tube (CAPP, Germany) with 45 µL of DMEM, 2% AA, and 2% FBS. This sample was tested to determine the transmission efficiency (TE) (the number of WNV‐positive saliva samples out of the total number of analyzed mosquitoes). Lastly, the mosquito’s head was added to the tube with its wings and legs. These body parts were tested to determine the dissemination efficiency (DE) (the number of mosquitoes with secondary tissues positive for WNV out of the total number of analyzed mosquitoes). The abdomen was added to a separate 2 mL EXPell Secure microcentrifuge tube with 500 µL DMEM and 2% AA and tested to determine the infection efficiency (IE) (the number of mosquitoes with WNV‐positive abdomens out of the total number of analyzed mosquitoes). All collected samples were stored at −80°C until analysis.

### 2.5. Viral RNA Extraction and RT‐qPCR Analysis

Silicon‐carbide sharp particles of 1 mm (Biospec Products, United States) were added to the samples containing abdomens and legs/wings/head for homogenization in the TissueLyser II (Qiagen, Germany) at 25 Hz for 6 min. Salivary samples were prepared by combining 25 µL of the sample with 115 µL of nuclease‐free water (Applied Biosystems, United States). RNA was extracted from all samples using the QIAamp viral RNA mini kit (Qiagen, Germany) according to the manufacturer’s protocol. In short, 140 µL of the supernatant of abdomen and legs/wings/head samples was added to 560 µL lysis buffer containing carrier RNA (diluted in an equal volume of elution buffer) in a 100:1 ratio. For the saliva samples, the prepared sample solution was used, and a synthetic RNA construct as an in‐house external extraction control was added in a 1:100 ratio to the mixture with lysis buffer and carrier RNA. After a 10‐minute incubation at room temperature, 560 µL of 96%–100% ethanol (Biosolve Chimie, France) was added, and the sample was transferred to the QIAamp mini column. After centrifugation for 1 min at 8000 rpm (centrifuge 5425, Eppendorf, Germany), the column was washed twice with 500 µL of two different wash buffers and loaded with 60 µL of elution buffer for RNA elution. The obtained RNA extracts were stored at −80°C until analysis by RT‐qPCR.

### 2.6. One‐Step RT‐qPCR

A WNV primer–probe mixture was prepared by combining 10 µL of each of the WNV NS2A primers (forward: 5′‐ CCT TTT CAG TTG GGC CTT CTG ‐3′ and reverse: 5′‐ ATC TTG GCY GTC CAC CTC TTG ‐3′, stock concentration of 100 µM) (IDT, United States), 4 µL of the WNV NS2A probe (5′‐ /56‐FAM/TTC TTG GCC /ZEN/ACC CAG GAG GTC/3IABkFQ/ ‐3′, stock concentration of 100 µM) (IDT, United States) [57], and 76 µL of nuclease‐free water. Additionally, each sample was tested for the housekeeping gene beta‐actin as an internal control. The beta‐actin primer–probe mixture consisted of 6 µL of both the forward and reverse universal actin primers (forward: 5′‐ CAG CAC AAT GAA GAT CAA GAT CAT C ‐3′ and reverse: 5′‐ CGG ACT CAT CGT ACT CCT GCT T ‐3′, stock concentration of 100 µM) (IDT, United States), 4 µL of the universal actin probe (5′‐ /5HEX/TCG CTG TCC /ZEN/ACC TTC CAG CAG ATG T /3IABkFQ/ ‐3′, stock concentration of 100 µM) (IDT, United States) [58], and 84 µL of nuclease‐free water. Lastly, a synthetic RNA construct was used as an external extraction control for the saliva samples. This control comprises a non‐WNV‐related RNA sequence and is prepared as described previously [59]. For the detection of the external control, a primer–probe mixture of 10 µL of both primers (forward: 5′‐ ATA AAT AAT CTG ACG TTT GTA ATG TCC GCT C ‐3′ and reverse: 5′‐ ATA AAT AAT CTC CAG TTG CTA CCG ATT TTA CAT A ‐3′, stock concentration of 100 µM) (IDT, United States), 2 µL of the probe (5′‐ /5TexRd‐XN/TTT GTA CCA CCT CCC ACC GAC CAT C/3IAbRQSp/ ‐3′, stock concentration of 100 µM) (IDT, United States) [59], and 78 µL of nuclease‐free water was used.

For each sample, one‐step RT‐qPCR reactions were run with the primer–probe combinations mentioned above. Each RT‐qPCR reaction mixture consisted of 12.5 µL of RT‐PCR buffer (2X) (AgPath‐ID One‐Step RT‐PCR Reagents kit, Applied Biosystems, United States), 1 µL of RT‐PCR enzyme mix (25X) (AgPath‐ID One‐Step RT‐PCR Reagents kit, Applied Biosystems, United States), 4.5 µL of nuclease‐free water, and 2 µL of the corresponding primer–probe mixture. In total, 20 µL of the different RT‐qPCR mixtures was distributed on a LightCycler 96‐well plate (Roche, Switzerland), to which 5 µL of sample RNA was added. The plate was covered with a LightCycler sealing foil (Roche, Switzerland) and centrifuged for one minute using a microcentrifuge (ClearLine, France). RT‐qPCR was performed on a LightCycler 480 (Roche, Switzerland) using the following temperature settings: 45°C for 10 min, 95°C for 10 min, 45 cycles at 95°C for 15 s and 60°C for 45 s, and a final step at 40°C for 30 s. The cut‐off for positivity was set at a Ct‐value of 40. Samples with a Ct‐value below 40 and with a characteristic exponential amplification curve were considered positive for WNV. Samples with a Ct‐value below 40 but without a clear exponential amplification curve in the one‐step RT‐qPCR were considered doubtful and tested in a two‐step RT‐qPCR for confirmation. When found positive in the two‐step RT‐qPCR, the Ct‐value of the one‐step RT‐qPCR was used for further analysis.

### 2.7. Two‐Step RT‐qPCR

Samples with a Ct‐value below 40 and without a clear exponential amplification curve in one‐step RT‐qPCR were tested in two‐step RT‐qPCR. First, the reverse transcription master mix was prepared by combining 4 µL of 5X first strand buffer (SuperScript III Reverse Transcriptase, Invitrogen, United States), 2 µL of 0.1 M DTT (SuperScript III Reverse Transcriptase, Invitrogen, United States), 2 µL of dNTP mix (10 mM) (Thermo Scientific, United States) diluted 1:2 in nuclease‐free water, 4 µL of random hexamers (Invitrogen, United States) diluted 1:10 in nuclease‐free water, 0.25 µL of RNaseOUT Ribonuclease Inhibitor (Invitrogen, United States), 0.5 µL of M‐MLV Reverse Transcriptase (Invitrogen, United States), and 3.25 µL of nuclease‐free water for each sample. Next, 16 µL of the master mix was distributed in the wells of a LightCycler 96‐well plate, and 4 µL of the sample RNA extract was added. The plate was sealed using a LightCycler sealing foil and centrifuged for 1 minute in a ClearLine microcentrifuge. Reverse transcription was performed on a LightCycler 480 with the following temperature program: 37°C for 1 h, 65°C for 10 min, and 40°C for 30 s. During the reverse transcription process, the master mix for the two‐step RT‐qPCR was prepared by combining 10 µL of the LightCycler 480 Probes Master (Roche, Switzerland), 2 µL of the premade primer–probe mixture (described in Section 2.6 One‐step RT‐qPCR), 0.32 µL of 25 mM MgCl_2_ (FastStart Taq DNA Polymerase kit, Merck, Germany), and 5.18 µL of nuclease‐free water per sample. Then, 17.5 µL of the master mix was added per well of a LightCycler 96‐well plate. When the reverse transcription was finished, 2.5 µL of cDNA was transferred to the plate containing the cDNA amplification master mix. The plate was sealed with a LightCycler sealing foil and centrifuged in a ClearLine microcentrifuge for 1 minute. cDNA samples were amplified on a LightCycler 480 under the following settings: 95°C for 5 min, 45 cycles at 95°C for 15 s and 60°C for 45 s, and 40°C for 30 s. Samples with a Ct‐value equal to or below 40 and with an exponential amplification curve were considered positive.

### 2.8. Species Determination Analysis

To identify the *Cx. pipiens* biotype, an additional RT‐qPCR reaction was performed on all abdomens. Universal *Cx. pipiens* primers targeting microsatellite CQ11 were used (forward: 5′‐ GCG GCC AAA TAT TGA GAC TTT C ‐3′ and reverse: 5′‐ ACT CGT CCT CAA ACA TCC AGA CAT A ‐3′) (IDT, United States) and were prepared at a stock concentration of 100 µM. To distinguish the biotype *pipiens*, probes Cpp_pip_P1 (5′‐ /56‐FAM/CAC ACA AAY /ZEN/CTT CAC CGA A/3IABkFQ/ ‐3′, stock concentration of 100 µM) and Cpp_pip_P2 (5′‐ /56‐FAM/ACA CAA ACC /ZEN/TTC ATC GAA /3IABkFQ/ ‐3′, stock concentration of 100 µM) were used, while probe Cpp_mol_P (5′‐ /56‐FAM/TGA ACC CTC /ZEN/CAG TAA GGT A/3IABkFQ/ ‐3′, stock concentration of 100 µM) (IDT, United States) was used to identify the *molestus* biotype [27]. First, a primer–probe mixture for each biotype was prepared. For the *pipiens* biotype, 3.85 µL of both the forward and reverse primers, 2.5 µL of the Cpp_pip_P1 probe, 1.25 µL of the Cpp_pip_P2 probe, and 88.55 µL of nuclease‐free water were combined. For the *molestus* biotype, 3.85 µL of both the forward and reverse primers, 1.25 µL of the Cpp_mol_P probe, and 91.05 µL of nuclease‐free water were combined. RT‐qPCR was performed for each biotype in separate reactions using a mixture containing 10 µL of Sso Advanced Universal Probes Supermix (Bio‐Rad Laboratories, United States), 2 µL of the corresponding primer–probe mixture, and 3 µL of nuclease‐free water per sample. Next, 15 µL of the master mix and 5 µL of the sample extract were added to a LightCycler 96‐well plate. The plate was sealed with a LightCycler sealing foil and centrifuged in a ClearLine microcentrifuge for 1 minute. RT‐qPCR was run on a LightCycler 480 with the following program: 95°C for 3 min and 45 cycles at 95°C for 15 s and 60°C for 45 s.

Samples that were not identified as either *Cx. pipiens pipiens* or *Cx. pipiens molestus* biotype were tested in an additional RT‐qPCR to identify for *Cx. torrentium*, a native Belgian species that is morphologically very close to *Cx. pipiens* [60]. An identical RT‐qPCR reaction as described above was performed but with the following primer–probe mixture: 3.85 µL of both primers (forward: 5′‐ CTT ATT AGT ATG ACA CAG GAC GAC AGA AA ‐3′ and reverse: 5′‐ GCA TAA ACG CCT ACG CAA CTA CTA A ‐3′, stock concentration of 100 µM), 1.25 µL of the probe (5′‐ /FAM/ATG ATG CCT GTG CTA CCA/MGB/ ‐3′, stock concentration of 100 µM) (IDT, United States), and 91.05 µL of nuclease‐free water [61].

### 2.9. Virus Isolation

To evaluate the presence of infectious WNV, all RT‐qPCR‐positive samples were tested for virus isolation. For abdomen and legs/wings/head samples, 50 µL of the homogenate was added to a 96‐well plate containing 80% confluent Vero cells. For saliva samples, 25 µL of each sample was mixed with 25 µL of DMEM. The cells with the inoculum were incubated at 37°C and 5% CO_2_ (CO_2_ incubator MCO‐170AICUVH‐PE, PHC Europe, the Netherlands) for 3 h, after which the supernatant was removed from the cells, and 100 µL of DMEM with 2% AA and 2% FBS was added. The cells were then incubated for 7 days at 37°C and 5% CO_2_. On day seven, a second passage was performed by transferring 50 µL of the supernatant from the first cell plate to a new 96‐well plate with 80% confluent Vero cells and by repeating the above‐described method. After the 7‐day incubation period, both plates were fixed by adding 100 µL cold methanol (Fisher Chemical, United States) and keeping them at −20°C for 20 min. After methanol removal, the plate was kept at −20°C without lid for at least 24 h. For WNV antigen visualization, 25 µL of a 1:200 dilution of the primary antibody anti‐WNV NS1 (Bio‐Techne, United States) in phosphate‐buffered saline (PBS) (Gibco, United States) was added to the cells and incubated for 1 h at 37°C and 5% CO_2_. After washing, 25 µL of a 1:200 dilution of the secondary antibody Alexa Fluor 488 goat anti‐mouse IgG (Invitrogen, United States) in PBS was added to the cells and incubated for 1 h at 37°C and 5% CO_2_. Lastly, cells were incubated for 3 min in the dark with 50 µL of a 1:1000 Hoechst dilution (Invitrogen, United States) in PBS before imaging with a Leica DM IL LED Fluo inverted microscope and the pE‐300^white^ LED fluorescent light source (Leica, Germany).

### 2.10. Statistical Analyses

Spearman’s rank correlation was used to analyze the correlation between the blood feeding rate and the number of mosquitoes exposed to the blood meal. A 3 × 2 chi‐square test (*Cx. pipiens*) and a 3 × 2 Fisher’s exact test (*Cx. torrentium*) was used to compare the IEs and DEs between the different temperature conditions. A 3 × 2 Fisher’s exact test was performed to compare the survival rates and TEs between the three temperature conditions. A 4 × 2 Fisher’s exact test was used to analyze the difference in IE, DE, and TE between the different *Cx. pipiens* biotypes, hybrids, and *Cx. torrentium* mosquitoes. The Mann–Whitney *U* test was used for comparing (i) the difference in Ct‐values of infection‐positive samples with and without dissemination for each temperature condition, (ii) the difference in Ct‐values of dissemination‐positive samples with and without transmission for each temperature, and (iii) the difference between the Ct‐values of isolation‐positive and ‐negative samples. The Kruskal–Wallis test was performed to analyze (i) the difference in Ct‐values of infection‐positive samples with and without dissemination and dissemination‐positive samples with and without transmission between the temperature conditions and (ii) the differences in Ct‐values between the *Cx. pipiens* biotypes, hybrids, and *Cx. torrentium*.

Chi‐square tests were used when 80% of all expected counts (based on the contingency table) were equal to or higher than five. If this criterion was not met, Fisher’s exact test was performed. The Shapiro–Wilk test was used for normality testing. In case of statistical differences in the 3 × 2 chi‐square test and the 4 × 2 Fisher’s exact test, the different temperature conditions or mosquito species/biotypes were compared two by two using the 2 × 2 chi‐square test or the 2 × 2 Fisher’s exact test as post hoc tests, respectively, together with the Bonferroni correction. Dunn’s test was used as a post hoc test for all analyses with the Kruskal–Wallis test.

Statistical analyses were performed in GraphPad Prism version 10.4.2, considering *p* < 0.05 as statistically significant. Graphs were created in GraphPad Prism, version 10.4.2.

## 3. Results

### 3.1. Blood Feeding and Survival Rates

#### 3.1.1. Blood Feeding Rates

**Figure 1:**
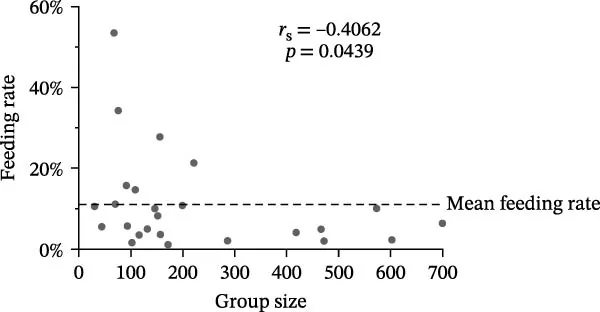
Association between mosquito group size and blood feeding rate. Each dot represents one feeding event and the dashed line indicates the overall mean blood feeding rate. The association was analyzed using Spearman’s rank correlation.

During the study, a total of 2876 female mosquitoes were exposed to an infectious blood meal during 25 feeding events. The average blood feeding rate was 11.1% across all blood feeding events in which at least one mosquito successfully fed, with a minimum and maximum blood feeding rate of 1.1% and 53.6%, respectively. A negative correlation (Spearman’s rank correlation; *p* = 0.0439, *r*_S_ = −0.4062) was found between the number of mosquitoes exposed to a blood meal and the blood feeding rate (Figure 1).

#### 3.1.2. Survival Rates

At 25°C, a total of 93.1% (54/58) of blood‐fed mosquitoes survived the 14‐day incubation period, while the survival at 25/15 and 25/20°C amounted to 89.3% (50/56) and 94.6% (70/74), respectively. No statistically significant differences (Fisher’s exact test; *p* = 0.5008) were observed in the survival rates between the three different temperature conditions.

### 3.2. Species Determination Analysis

**Figure 2:**
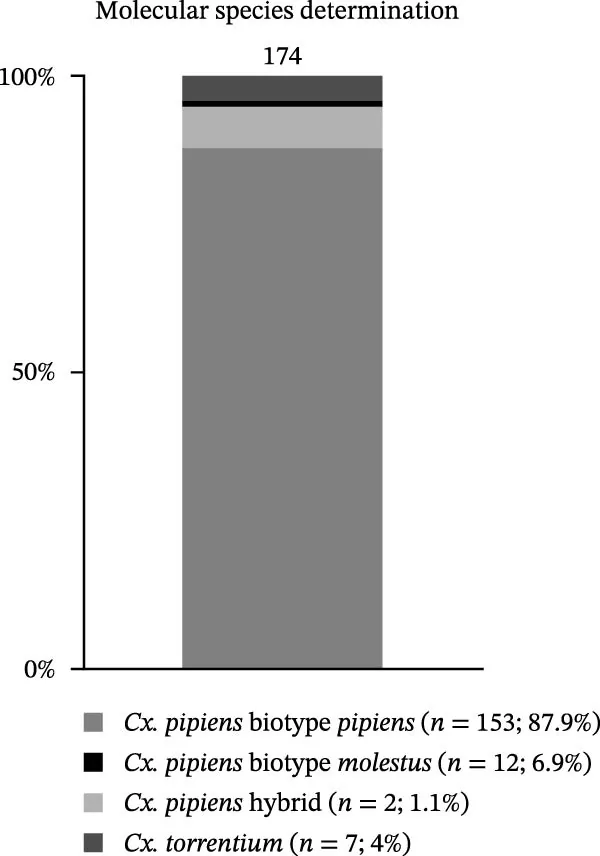
Overview of the molecular species identification by RT‐qPCR. Bar represents the percentage of mosquitoes identified as *Cx. pipiens* biotype *pipiens*, *Cx. pipiens* biotype *molestus*, *Cx. pipiens* hybrids, and *Cx. torrentium*. The total number of all tested mosquitoes is shown above the bar, and absolute numbers and percentages per species/biotype are mentioned in the legend.

Species identification by RT‐qPCR showed that the majority (*n* = 153/174; 87.9%) of mosquitoes that were blood fed and survived the 14‐day incubation period was *Cx. pipiens* biotype *pipiens*, while only 12 (6.9%) belonged to the biotype *molestus*. Two (1.1%) of the mosquitoes were hybrid. The remaining seven mosquitoes (4%) were identified as *Cx. torrentium* (Figure 2).

### 3.3. Infection, Dissemination, and TEs at Different Temperature Conditions for *Cx. pipiens*

In this section, the results of *Cx. pipiens* biotype *pipiens* and biotype *molestus* and the *Cx. pipiens* hybrids obtained in this study are described.

#### 3.3.1. IEs

**Figure 3:**
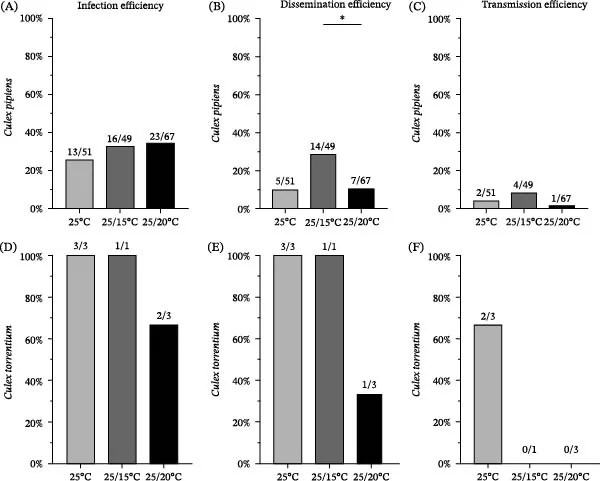
Infection efficiencies (A, D), dissemination efficiencies (B, E), and transmission efficiencies (C, F) of WNV in Belgian field‐collected *Cx. pipiens* (A–C) and *Cx. torrentium* (D–F) mosquitoes at three different temperature conditions and after a 14‐day incubation period. Bars represent the percentage of mosquitoes that tested positive for WNV with RT‐qPCR. Absolute numbers are mentioned above each bar. Differences in *Cx. pipiens* and *Cx. torrentium* efficiencies between the temperature groups were evaluated using chi‐square and Fisher’s exact statistical tests, and the Bonferroni correction was applied to the significant *p*‐values when multiple two‐by‐two comparisons were done.  ^∗^ *p* = 0.0123.

RT‐qPCR revealed that 13 out of 51 blood‐fed mosquitoes tested positive for WNV in their abdomen at the 25°C temperature condition, corresponding to an IE of 25.5%. At 25/15°C, *Cx. pipiens* mosquitoes showed an IE of 32.7% (16/49). Lastly, an IE of 34.3% (23/67) was obtained at a 25/20°C temperature gradient. No statistically significant differences (chi‐square test; *p* = 0.5686) were observed between the IEs at the three tested temperatures (Figure 3A).

#### 3.3.2. DEs

At 25°C, RT‐qPCR showed that five out of 51 mosquitoes with a positive IE had WNV RNA in their legs/wings/head, leading to a DE of 9.8%. At 25/15°C, 28.6% (14/49) of mosquitoes tested WNV RNA‐positive in their legs/wings/head. Interestingly, this included five mosquitoes that were infection‐positive and nine infection‐negative. Seven out of 67 (10.4%) mosquitoes were positive for WNV dissemination at 25/20°C. Here, four were infection‐positive, while the remaining three were negative for infection. The DE at 25/15°C was significantly higher than that at 25/20°C (chi‐square test; *p* = 0.0123), and just not significantly higher than the DE observed at 25°C (chi‐square test; *p* = 0.0168, which exceeds the Bonferroni‐corrected alpha‐value of 0.0167) (Figure 3B).

#### 3.3.3. TEs

In total, two out of 51 mosquitoes were WNV RNA‐positive in the saliva at 25°C, leading to a TE of 3.9%. Four out of 49 mosquitoes had WNV‐positive saliva at 25/15°C, corresponding to a TE of 8.2%. At 25/20°C, a TE of 1.5% (1/67) was obtained. All transmission‐positive mosquitoes were also dissemination‐positive at 14 days post‐blood feeding. Statistical analysis did not show significant differences (Fisher’s exact test; *p* = 0.2317) between the TEs at all tested temperature conditions (Figure 3C).

### 3.4. Infection, Dissemination, and TEs at Different Temperature Conditions for *Cx. torrentium*

A limited number of mosquitoes used in this study were identified as *Cx. torrentium*. The following paragraph describe the observed IEs, DEs, and TEs for this species at the three tested temperatures.

At 25°C, all *Cx. torrentium* mosquitoes (3/3) were positive for infection as well as at 25/15°C (1/1). Two out of three mosquitoes were positive for infection by RT‐qPCR at 25/20°C. All infection‐positive mosquitoes were also positive for dissemination at 25°C (3/3) and 25/15°C (1/1). At 25/20°C, one out of three mosquitoes was positive for WNV in the legs/wings/head. Lastly, transmission‐positive mosquitoes were only found at 25°C (2/3) (Figure 3).

### 3.5. Viral Loads for Infection, Dissemination, and Transmission in *Cx. pipiens*

**Figure 4:**
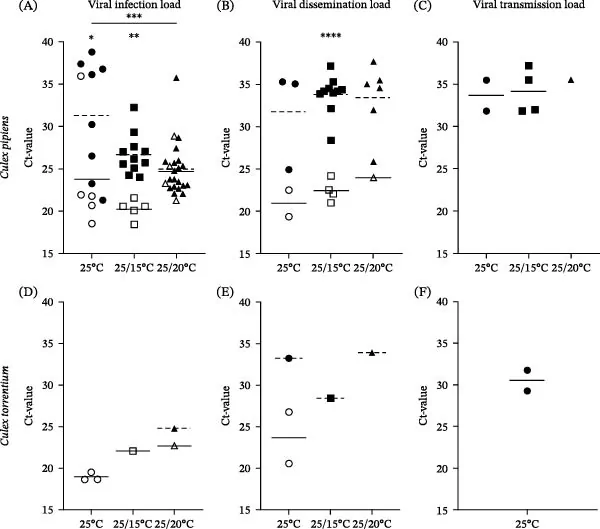
WNV loads (Ct‐values) in abdomens (A, D), legs/wings/head (B, E), and saliva (C, F) of blood‐fed *Cx. pipiens* (A–C) and *Cx. torrentium* (D–F) mosquitoes held at three temperature conditions during a 14‐day period. Open symbols in A/D and B/E indicate mosquitoes that have tested positive for WNV in dissemination and transmission, respectively. Filled symbols in A/D and B/E indicate mosquitoes that tested negative for WNV in dissemination and transmission, respectively. Full and dashed horizontal lines indicate the mean Ct‐values of samples with and without dissemination (A, D), and with and without transmission (B, E), respectively. Horizontal lines in C and F indicate the mean Ct‐values. The Mann–Whitney *U* test was used to analyze differences in Ct‐values between infection‐positive samples with and without dissemination and dissemination‐positive samples with and without transmission for each temperature condition. The Kruskal–Wallis test was used to compare the difference in Ct‐value of infection‐positive samples with dissemination between the temperature conditions, as well as for those infection‐positive samples without dissemination and dissemination‐positive samples with and without transmission.  ^∗^ *p* = 0.0451,  ^∗∗^ *p* < 0.0001,  ^∗∗∗^ *p* = 0.0020, and  ^∗∗∗∗^ *p* = 0.0020.

At 25 and 25/15°C, the viral loads (based on Ct‐values) of positive infection samples with WNV dissemination were significantly higher than those without WNV dissemination (Mann–Whitney *U* test; *p* = 0.0451 and *p* < 0.0001, respectively) (Figure 4A). At 25/20°C, no significant differences (Mann–Whitney *U* test; *p* = 0.9687) were observed between the viral loads of infection‐positive samples with and without dissemination. Similarly, the viral loads in dissemination‐positive samples with positive saliva were significantly higher (Mann–Whitney *U* test; *p* = 0.0020) than in those with negative saliva at 25/15°C (Figure 4B). This difference was not significant (Mann–Whitney *U* test; *p* = 0.2000) at the 25°C condition. The significance at 25/20°C could not be analyzed as only one mosquito tested positive for both dissemination and transmission. At all three temperature conditions, the transmission‐positive mosquitoes had the highest viral dissemination loads (Figure 4C).

Viral loads of infection‐positive samples with positive dissemination were not significantly different between the different temperatures, while the viral loads of samples positive for infection and negative for dissemination were significantly lower at 25°C than at 25/20°C (Kruskal–Wallis test; *p* = 0.0020) (Figure 4A). Statistical analysis did not show any differences (Kruskal–Wallis test; *p* = 0.6667) in the viral loads of dissemination‐positive samples with positive transmission between the different temperature conditions, as well as for dissemination‐positive samples with negative transmission (Kruskal–Wallis test; *p* = 0.8057).

### 3.6. Viral Loads for Infection, Dissemination, and Transmission in *Cx. torrentium*

All infection‐positive mosquitoes showed low Ct‐values (<25) after RT‐qPCR analysis. Therefore, the majority of mosquitoes were also positive for WNV dissemination, with some showing higher Ct‐values than others. Those dissemination‐positive mosquitoes with the lowest Ct‐values were also positive for transmission (Figure 4D–F).

### 3.7. Virus Isolation of *Cx. pipiens*

All *Cx. pipiens* samples that tested positive for WNV with RT‐qPCR were subjected to virus isolation. In total, 65.9% (56/85) of the samples were confirmed to contain infectious WNV. The Ct‐values of WNV isolation‐positive samples were significantly lower than those of isolation‐negative samples (Mann–Whitney *U* test; *p* < 0.0001). Importantly, all RT‐qPCR‐positive saliva samples at the 25°C temperature condition and three out of four saliva samples at the 25/15°C gradient were found positive in virus isolation, confirming the presence of infectious virus in the saliva. For the 25/20°C gradient, the one RT‐qPCR‐positive saliva sample remained negative for virus isolation (Table 1).

**Table 1:** Percentages of RT‐qPCR‐positive samples of *Cx. pipiens* that were confirmed by virus isolation.

Temperature condition	Infection samples	Dissemination samples	Transmission samples
25°C	46.2% (6/13)	60% (3/5)	100% (2/2)
25/15°C	68.8% (11/16)	57.1% (8/14)	75% (3/4)
25/20°C	91.3% (21/23)	28.6% (2/7)	0% (0/1)

*Note:* Efficiencies are shown in percentages and absolute numbers are mentioned in brackets.

### 3.8. Comparison Between *Cx. pipiens* and *Cx. torrentium*

**Figure 5:**
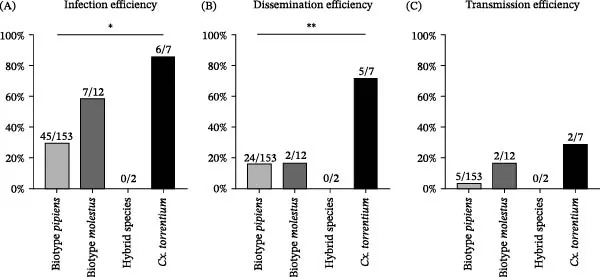
Overall IE (A), DE (B), and TE (C) of all mosquitoes analyzed in the vector competence study, expressed per biotype or species. Bars represent the percentage of mosquitoes that tested positive for WNV with RT‐qPCR. Absolute numbers are mentioned above each bar. Differences between the species/biotypes were evaluated using Fisher’s exact statistical test, and the Bonferroni correction was applied to the significant p‐values.  ^∗^ *p* = 0.0045,  ^∗∗^ *p* = 0.0023.

The IEs, DEs, and TEs obtained at the three temperature conditions were combined and split over the different *Cx. pipiens* biotypes/hybrids and *Cx. torrentium* to evaluate potential biotype or species‐specific differences (Figure 5). *Cx. torrentium* had the highest IE, DE, and TE. Both the IE (Fisher’s exact test; *p* = 0.0045) and DE (Fisher’s exact test; *p* = 0.0023) of *Cx. torrentium* were significantly higher compared to those of *Cx. pipiens* biotype *pipiens* (Figure 5A,B). No statistical differences were found for the IE, DE, and TE (Figure 5C) between both *Cx. pipiens* biotypes.

Statistical analyses also showed that the Ct‐values of infection‐positive *Cx. torrentium* samples were significantly lower than those of *Cx. pipiens* biotype *pipiens* (Kruskal–Wallis test; *p* = 0.0311). No significant differences were found between the Ct‐values obtained at infection, dissemination, and transmission for the other species/biotypes.

Virus isolation confirmed that three out of five (60%) WNV RT‐qPCR‐positive saliva samples of *Cx. pipiens* biotype *pipiens* were positive, as well as all WNV RT‐qPCR‐positive saliva samples of *Cx. pipiens* biotype *molestus* (*n* = 2; 100%) and *Cx. torrentium* (*n* = 2; 100%). Both hybrid mosquitoes were negative for infection, dissemination, and transmission. Full details for each temperature condition can be found in Table S1.

## 4. Discussion

Urbanization, globalization, and climate change allow pathogens to be introduced into new regions [1, 8]. WNV has been spreading throughout Europe over the last few decades and can be considered an emerging pathogen. Although Belgium has not yet reported locally acquired human or equine infections, the recent detection of WNV in wild birds confirms that the virus has entered the country and may establish local transmission. In this context, we studied whether Belgian *Cx. pipiens* mosquitoes are competent vectors for WNV under different temperature conditions and thus could play an important role in WNV transmission upon introduction. This species is considered to be an important vector of WNV as it was found to be competent in neighboring countries [38, 41, 44] and as it is the most abundant mosquito species in Belgium [62].

This study was conducted with adult mosquitoes that had emerged from field‐collected larvae. The use of these larvae leads to highly relevant results as they most closely represent natural mosquito populations [63]. However, the use of field‐collected larvae also comes with important challenges. First of all, collecting the larvae was a time‐intensive process due to their limited yearly availability and dependance on rainfall. A second challenge encountered was the low blood feeding rate. Certain mosquito species have higher overall blood feeding rates compared to other species, and blood feeding success can depend on the use of anesthetized animals or artificial blood feeding systems. Additionally, blood feeding rates can differ between field‐collected mosquitoes and the laboratory colony. In this study, a low average blood feeding rate of 11.1% was obtained from the F0‐generation *Cx. pipiens* mosquitoes. The mean blood feeding rate of 20.8% was reported in another membrane‐feeding experiment with field‐collected adult *Cx. pipiens* mosquitoes [28] is higher than that obtained here, yet still markedly lower than that typically observed for highly anthropophilic species such as *Aedes aegypti* [64]. Our low feeding success is likely related to the use of F0‐mosquitoes that were not adapted to laboratory conditions and using the Hemotek membrane‐feeding system instead of direct host feeding [65]. Contrary to these challenges, the *Cx. pipiens* mosquitoes in our study had a high average 14‐day survival rate of 92.3%, which is comparable to or higher than those reported in other studies [28, 40].

In this study, we demonstrated that Belgian *Cx. pipiens* mosquitoes are competent vectors for WNV at 25°C, with an IE, DE, and TE of 25.5%, 9.8%, and 3.9%, respectively. This finding was confirmed by virus isolation, as 100% of the RT‐qPCR‐positive saliva samples were also positive in isolation. Previous studies have assessed the vector competence of *Cx. pipiens* for WNV under constant temperature conditions. French *Cx. pipiens* mosquitoes infected with WNV showed a DE of 38.5% and a TE of 3.8% [38]. In Germany, IEs of approximately 28% were observed for *Cx. pipiens* biotype *pipiens* and 48% for *Cx. pipiens* biotype *molestus* was reported [ 41], while *Cx. pipiens* mosquitoes in the Netherlands had an IE of 49% and a TE of 22% [40]. A previous Belgian study reported an IE of 10.6% and a DE and TE of 4.3% [28]. In general, these studies, including our data, conclude that *Cx. pipiens* mosquitoes are able to transmit WNV at a constant temperature. Individual differences between our results and those reported in the studies mentioned above can probably be explained by variations in the viral titer in the blood meal, incubation temperature, working with laboratory‐reared mosquitoes compared to those of field‐collected mosquitoes, and genetic factors that might vary among mosquitoes from different geographical areas. We used a low but relevant WNV titer of 10^5.7^ TCID_50_/mL in the blood meal, which is lower than the titer used in the other studies mentioned above and might therefore explain the lower TE found in our study compared to that in others. This titer was selected as it has been shown that a viremia of 1.4 × 10^4^–1.4 × 10^5^ TCID_50_/mL is sufficient to infect feeding *Culex* mosquitoes [66]. Additionally, a study by Goddard et al. [67] reported that *Cx. pipiens* infected with a WNV titer of 1 log lower than that used in our study were positive for transmission [67].

The incubation at a constant temperature as described above is not representative of the conditions at which mosquitoes are exposed in the field in Belgium. Therefore, we next determined the vector competence of Belgian *Cx. pipiens* mosquitoes for WNV at a temperature gradient of 25°C during daytime and 15°C during nighttime, representative of current summer temperatures. Our results showed an IE, DE, and TE of 32.7%, 28.6%, and 8.2%, respectively. Virus isolation confirmed 75% of WNV‐positive saliva samples, indicating that Belgian *Cx. pipiens* mosquitoes are competent for WNV at this temperature gradient. The efficiencies obtained at this temperature condition were not significantly different from those found at the 25°C condition, suggesting that a lower night temperature does not impact the midgut and salivary gland barriers within the mosquito and therefore the vector competence. This finding aligns with a study comparing the effect of static and fluctuating temperatures on WNV infections in *Cx. quinquefasciatus* and *Cx. tarsalis* [68]. However, based on results previously observed by Van Den Eynde et al. [69], it would be expected that more successful infections would have been obtained at 25°C compared to 25/15°C. The authors reported a significantly higher DE at 25°C compared to 25/15°C for Japanese encephalitis virus, a flavivirus similar to WNV, in Belgian *Cx. pipiens* mosquitoes. This result suggests that fluctuating temperatures negatively impact the midgut barrier, making it more difficult for the Japanese encephalitis virus to pass through it [69]. However, WNV appears to be relatively insensitive to these thermal fluctuations, implying that restrictions to pass the midgut barrier are virus‐specific. There is also some evidence that fluctuating temperatures increase the WNV titer in *Cx. tarsalis* mosquitoes, while viral titers in *Cx. quinquefasciatus* was increased at static temperatures [68]. These findings indicate possible interspecies differences and might explain our observed results for *Cx. pipiens* and WNV.

As climate change (rising temperatures and altered thermal patterns) can influence the vector competence of mosquitoes for WNV, an additional temperature gradient of 25°C during daytime and 20°C during nighttime was assessed to simulate a climate change scenario. At this temperature condition, we reported an IE of 34.3%, a DE of 10.4%, and a TE of 1.5%. Virus isolation did not confirm the only RT‐qPCR‐positive saliva sample, making that we cannot state with certainty that *Cx. pipiens* mosquitoes would be able to transmit WNV under these conditions. The number of mosquitoes tested is; however, too low to rule out this possibility with certainty. While the IE and TE, as determined by RT‐qPCR, are similar compared to those at 25 and 25/15°C, the DE at 25/15°C was higher than in both other conditions, and this difference was statistically significant compared to the 25/20°C condition. This finding suggests that WNV is more restricted in passing the midgut barrier when night temperatures only drop to 20°C instead of 15°C. Although it needs to be proven, a hypothesis could be that the immune mechanisms that limit WNV spread through the midgut barrier, for example, RNAi, are impaired at lower temperatures [70], leading to a decreased resistance against WNV invasion of the mosquito’s midgut, allowing the virus to pass more readily through the midgut barrier at 25/15°C. Despite the difference in DE, our results did not show a difference in TEs between 25/15 and 25/20°C, suggesting that the midgut barrier is more sensitive to temperature fluctuations than the salivary gland barrier. Under this climate change scenario, as well as at 25/15°C, we observed some dissemination‐positive mosquitoes that were negative for infection, which is related to the 14‐day incubation period. This finding indicates that WNV kinetics might differ between individual mosquitoes and vary with temperature. Additional research can provide more insight into the mechanism underlying this observation.

To gain additional insight into the within‐mosquito dynamics underlying these temperature effects, we analyzed the viral loads (Ct‐values) in infection, dissemination, and transmission samples. Similar to previous observations [71], the viral loads in our study were significantly higher for infection‐positive samples with WNV dissemination than those without dissemination at 25 and 25/15°C, indicating that crossing the midgut barrier depends on the viral load resulting from viral replication in the midgut. No such significant difference was observed at the 25/20°C condition, mostly due to the fact that the Ct‐values found in the infection‐positive but dissemination‐negative mosquitoes were lower than those in the other temperature conditions. Further research will have to show whether this specific temperature condition causes altered replication kinetics within the mosquito or differently impacts mechanical or immunological barriers hindering the crossing of the midgut barrier, even at high viral loads. Dissemination‐positive samples with transmission showed statistically higher viral loads at 25/15°C than those without transmission, as previously described [72]. Even though no such significant differences were found at the 25 and 25/20°C conditions, most likely due to the limited number of samples, transmission‐positive mosquitoes at these conditions were also those with the highest viral dissemination loads. This observation indicates that a sufficiently high viral load in secondary tissues may be necessary to cross the salivary gland barrier and result in successful transmission.

Seen that the vector competence can vary substantially between *Culex* species and between the *pipiens* and *molestus* biotypes of *Cx. pipiens*, we additionally assessed the species/biotype identity of all mosquitoes tested in this study. Vector competence comparisons between species were performed without taking into account the temperature conditions, and the results should therefore be interpreted carefully as temperature can affect vector competence. RT‐qPCR identification revealed a ratio of 87.9% of the biotype *pipiens* and 6.9% of the biotype *molestus*, which is as expected when working with Belgian, field‐collected larvae [60]. No statistical differences were observed in infection, dissemination, and transmission between both *Cx. pipiens* biotypes, implying that both biotypes are equally competent in transmitting WNV. These findings were also reported in other studies [41, 73]. However, some studies observed a higher vector competence of *Cx. pipiens* biotype *pipiens* [28, 74], while others state that the *molestus* biotype is the most competent [39]. Hybrid species between both biotypes represented 1.1% of all tested mosquitoes, which were all negative for infection, dissemination, and transmission. Besides, the *Cx. pipiens* biotypes, and 4% of all mosquitoes was identified as *Cx. torrentium*. This confirms the recent observation that *Cx. torrentium* co‐occurs with *Cx. pipiens* biotype *pipiens* and *molestus* in Belgium [75]. *Cx. torrentium* appeared to be a highly competent vector for WNV, as high infection, dissemination, and TEs were obtained in our study. Although the absolute numbers tested were limited, and the results should thus be interpreted with care, the IE and DE of *Cx. torrentium* were significantly higher compared to those of *Cx. pipiens* biotype *pipiens*. Studies using Finnish and German populations of *Cx. torrentium* and *Cx. pipiens* biotype *pipiens* have obtained similar results [34, 76] and thus indicate that *Cx. torrentium* could contribute to the spread of WNV upon its introduction in Belgium.

We showed that Belgian field‐collected *Cx. pipiens* mosquitoes are competent for WNV under different temperature conditions. It is therefore expected that they will play an important role in WNV transmission under field conditions, certainly since this mosquito species is highly abundant in Belgium [62] and prefers to feed on birds [44]. Therefore, even modest TEs can sustain enzootic WNV circulation when mosquito densities and bird‐host availability are high. From this, *Cx. pipiens* can be considered a high‐capacity vector for WNV in Belgium.

## 5. Conclusion

WNV continues to spread across Europe and poses challenges from a One Health perspective. In this study, the vector competence of Belgian, field‐collected *Cx. pipiens* mosquitoes was proven under a constant temperature of 25°C and under current Belgian summer temperatures (25/15°C). This, together with its high abundance and host preferences, suggests that this species could play an important role in the local spread of WNV upon its introduction in Belgium. Its vector competence under a climate change scenario (25/20°C) could not be confirmed as the RT‐qPCR‐positive saliva sample could not be confirmed via virus isolation. Our results furthermore showed that different temperature conditions impact the transmission of WNV through the midgut and highlight the importance of future studies to unravel the underlying mechanistic processes. Our findings underline that Belgium should be prepared for the possibility of local WNV circulation and indicate that an enzootic transmission cycle could establish rapidly following virus introduction. Continuous, integrated surveillance of birds, horses, and mosquitoes will therefore remain essential to detect early circulation.

## Author Contributions

**Evi Brams:** conceptualization, methodology, formal analysis, investigation, data curation, writing – original draft, writing – review and editing, visualization, project administration. **Charlotte Sohier**: conceptualization, writing – review and editing, supervision. **Nick De Regge**: conceptualization, resources, writing – review and editing, supervision, funding acquisition.

## Funding

This research has received funding from the Belgian Science Policy Office (BELSPO) (Research Project B2/223/P1/MODEVECO).

## Disclosure

All authors have read and agreed to the published version of the manuscript.

## Ethics Statement

The study was conducted using mosquitoes. In accordance with institutional and national guidelines, research involving insects does not require ethical approval. As such, no ethical approval was obtained.

## Conflicts of Interest

The authors declare no conflicts of interest.

## Supporting Information

Additional supporting information can be found online in the Supporting Information section.

## Supporting information


**Supporting Information** Table S1: Overview of the infection, dissemination, and transmission efficiencies per temperature condition for all mosquito biotypes/species analyzed in the vector competence study. Efficiencies are shown in percentages, and absolute numbers are mentioned in brackets.

## Data Availability

All data supporting our findings are included in the manuscript or Supporting Information.
