# Supplementary material for: Field‐Collected Belgian Culex pipiens and Culex torrentium Mosquitoes are Competent for West Nile Virus Under Different Temperature Conditions
Source: Transbound Emerg Dis. 2026 Sep 21;2026:4759838. doi: 10.1155/tbed/4759838 (PMC13594763; doi:10.1155/tbed/4759838)
Supplement: Supplementary file 1 [file TBED-2026-4759838-s001.docx]

Table 1. Overview of the infection, dissemination, and transmission efficiencies per temperature condition for all mosquito biotypes/species analyzed in the vector competence study. Efficiencies are shown in percentages, and absolute numbers are mentioned in brackets.

|  |  | ***Cx. pipiens* biotype *pipiens*** | ***Cx. pipiens* biotype *molestus*** | **Hybrid species** | ***Cx. torrentium*** |
| --- | --- | --- | --- | --- | --- |
| **25°C** | **Absolute percentage** | 88.9% (48/54) | 5.6% (3/54) | 0% (0/54) | 5.6% (3/54) |
|  | **Infection efficiency** | 22.9% (11/48) | 66.7% (2/3) | 0% (0/0) | 100% (3/3) |
|  | **Dissemination efficiency** | 8.3% (4/48) | 33.3% (1/3) | 0% (0/0) | 100% (3/3) |
|  | **Transmission efficiency** | 2.1% (1/48) | 33.3% (1/3) | 0% (0/0) | 66.7% (2/3) |
| **25/15°C** | **Absolute percentage** | 94% (47/50) | 2% (1/50) | 2% (1/50) | 2% (1/50) |
|  | **Infection efficiency** | 31.9% (15/47) | 100% (1/1) | 0% (0/1) | 100% (1/1) |
|  | **Dissemination efficiency** | 27.7% (13/47) | 100% (1/1) | 0% (0/1) | 100% (1/1) |
|  | **Transmission efficiency** | 6.4% (3/47) | 100% (1/1) | 0% (0/1) | 0% (0/1) |
| **25/20°C** | **Absolute percentage** | 82.9% (58/70) | 11.4% (8/70) | 1.4% (1/70) | 4.3% (3/70) |
|  | **Infection efficiency** | 32.8% (19/58) | 50% (4/8) | 0% (0/1) | 66.7% (2/3) |
|  | **Dissemination efficiency** | 12.1% (7/58) | 0% (0/8) | 0% (0/1) | 33.3% (1/3) |
|  | **Transmission efficiency** | 1.7% (1/58) | 0% (0/8) | 0% (0/1) | 0% (0/3) |
